# Nutritional Value and Antioxidant Potential of Djiboutian Abundant Seaweeds, With Their Food Applications in Doughnut and Tartare

**DOI:** 10.1002/fsn3.70956

**Published:** 2025-09-16

**Authors:** Moustapha Nour, Valérie Stiger‐Pouvreau, Abdourahman Daher, Solène Connan, Ahmed Ali, Louna Marchand, Matthieu Waeles, Sylvain Petek

**Affiliations:** ^1^ Centre d'Études et de Recherche de Djibouti Institut des Sciences de la Vie ISV Djibouti City Djibouti; ^2^ Univ Brest, IRD, CNRS, Ifremer, LEMAR, IUEM Plouzane France

**Keywords:** algal flour and flakes, Djiboutian macroalgae, functional food, ORAC, polyphenols, trace element

## Abstract

This study evaluated the nutritional composition and potential food applications of marine macroalgae from the Tadjourah Gulf (Djibouti), including brown (*Padina pavonica, Sargassum ilicifolium, S* . *latifolium* *, Turbinaria decurrens*), green (*Ulva clathrata*), and red ( *Hypnea musciformis* ) species. Analyses focused on their proximate composition, bioactive phenolics, mineral and trace elements, heavy metal content, and recommended daily Allowance (RDA). *Hypnea* and *Ulva* were rich in carbohydrates, while *Turbinaria* exhibited high amino acid levels. *Padina* stood out for its high chlorophyll *a, c*, carotenoids, phenolic content, and associated antioxidant activities. It also provided significant amounts of essential minerals, with RDA > 15% for calcium, magnesium, phosphorus, iron, manganese, copper, and zinc, positioning it as a valuable source for nutritional supplementation. All species complied with regulatory standards for heavy metal content, ensuring their safety for food consumption. Sensory evaluations highlighted their culinary potential in traditional dishes like doughnuts (seaweed flour) and tartare (seaweed flakes), with *Hypnea, Ulva*, *Padina*, and *S*. *ilicifolium* showing promising results. These findings positioned Djiboutian seaweeds as novel ingredients in traditional and modern food recipes and contributed to developing new supplements, recipes, and functional foods to diversify food in Djibouti. Moreover, it could strengthen food security in Djibouti, where addressing nutritional challenges is critical.

## Graphical abstract

This study explores the untapped potential of Djiboutian macroalgae as food ingredients, focusing on their nutritional composition (Proteins, amino acids and carbohydrates, including mannitol), phenolic content, and associated antioxidant properties. We also assess the recommended daily allowance of these macroalgae and present the results of a blind tasting test for two algae‐based food formulations. Our findings demonstrate the promising nutritional and functional benefits of macroalgae, supporting their use in developing sustainable, health‐promoting food products.

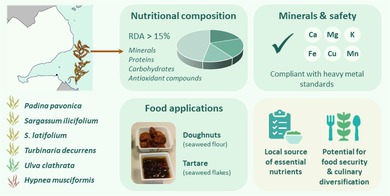

## 1 Introduction

Considering the escalating challenges posed by global population growth and environmental degradation, using natural bioresources has emerged as an essential means of addressing pressing concerns about freshwater scarcity and desertification (Tiwari and Troy 2015). In this context, the UN's Agenda 2030 and the EU's Biodiversity Strategy 2030 promote sustainable practices in agriculture and industry (European Commission 2021, 2023). In response to these challenges, the cultivation of macroalgae could provide some solutions.

With around 12,159 marine macroalgal species identified (Guiry 2024), their growing role in various sectors such as food, fertilizers, and plant biostimulants is increasingly recognized (FAO 2021). The consumption of seaweed has been a traditional practice in Asian countries for centuries. However, it is now gaining attention in the Western world for its recognized nutritional value and health benefits (Cian et al. 2015). On average, algae contain a wide range of metabolites, such as polyphenols (1%–25% DW), carbohydrates (20%–50% DW), proteins (5%–47% DW), lipids (1%–5% DW), polysaccharides (70% DW), carotenoids, and minerals (8%–40% DW) that greatly enhance their nutritional value (Barba 2017; Fakayode et al. 2020; MacArtain et al. 2008; Véliz et al. 2023).

Compounds derived from algae have been widely studied for their antioxidant, antibacterial, and anticancer activities and for preventing diseases associated with aging and obesity (Jesumani et al. 2019; Cao et al. 2020; López‐Hortas et al. 2021; Ismail, El Zokm, Sikaily, et al. 2023; Ismail, El Zokm, and Miranda Lopez 2023; Magwaza and Islam 2023; Bayomy and Alamri 2024). Because of these beneficial properties, they have applications in the food, nutraceutical, and cosmetics industries. The properties of algae are influenced by external environmental factors such as salinity, nutrient availability, light, depth, and seasonal variations of these, and they are also affected by intrinsic factors including the algal species, age, size, and tissue type (Lopez‐Santamarina et al. 2020). Various studies have shown that incorporating a certain proportion of seaweed powder contributes to the final product's nutritional quality, as indicated in several works with the incorporation of seaweed flakes in Camembert or yoghurt (Hell et al. 2018; Rioux et al. 2017; Lafeuille et al. 2023; Rodríguez et al. 2024), or with the incorporation of *Ulva* (Mohibbullah et al. 2023) and *Caulerpa* (Ngadiarti et al. 2022) flakes to make cookies.

A hot, arid climate, limited water resources, and a nomadic way of life characterize Djibouti. As a result, agricultural activity remains relatively limited, contributing to a high level of dependence on food imports from neighboring countries (Gouvernement de Djibouti 2000). In addition, a widespread lack of reliable access to food, characterized by a significant reliance on cereal, oil, and sugar‐rich foods, coupled with low meat consumption, is a key factor contributing to the high prevalence of malnutrition, estimated between 15% and 20% across various regions (WFP 2013). Djibouti has 372 km of maritime coastline and over 7000 km^2^ of maritime space and harbors an array of marine algae. Concerning macroalgae, the dominant genera within this ecosystem include *Hypnea, Padina*, *Sargassum*, *Turbinaria*, and *Ulva* (Government of Djibouti 2000). Despite a lack of available data on macroalgae from Djibouti, a few studies have begun to examine their potential to adsorb metals from wastewater and their antioxidant properties (Aden et al. 2022; Fourreh et al. 2019; Nour et al. 2024).

As part of the diversification of Djibouti's food sources, we studied the biochemical composition, mineral content, and determination of specific heavy metals in abundant macroalgae found in the Gulf of Tadjourah. Furthermore, the phenolic extract's antioxidant potential was investigated to enhance its applicability in nutrition, food, and nutraceuticals. Interestingly, we carried out a tasting test with the aim of testing if the incorporation of algae in two preparations—a doughnut made with macroalgal flour, which is a traditional meal in Djibouti, and a tartare using seaweed flakes following a traditional recipe from Brittany (France). The study demonstrated the potential of Djibouti seaweeds to serve as a food nutrient or in supplementation, with a daily intake percentage that aligns with the recommended dietary allowance (RDA) for several essential nutrients.

## 2 Materials and Methods

### 2.1 Sampling Locations and Seaweed Collection

**FIGURE 1:**
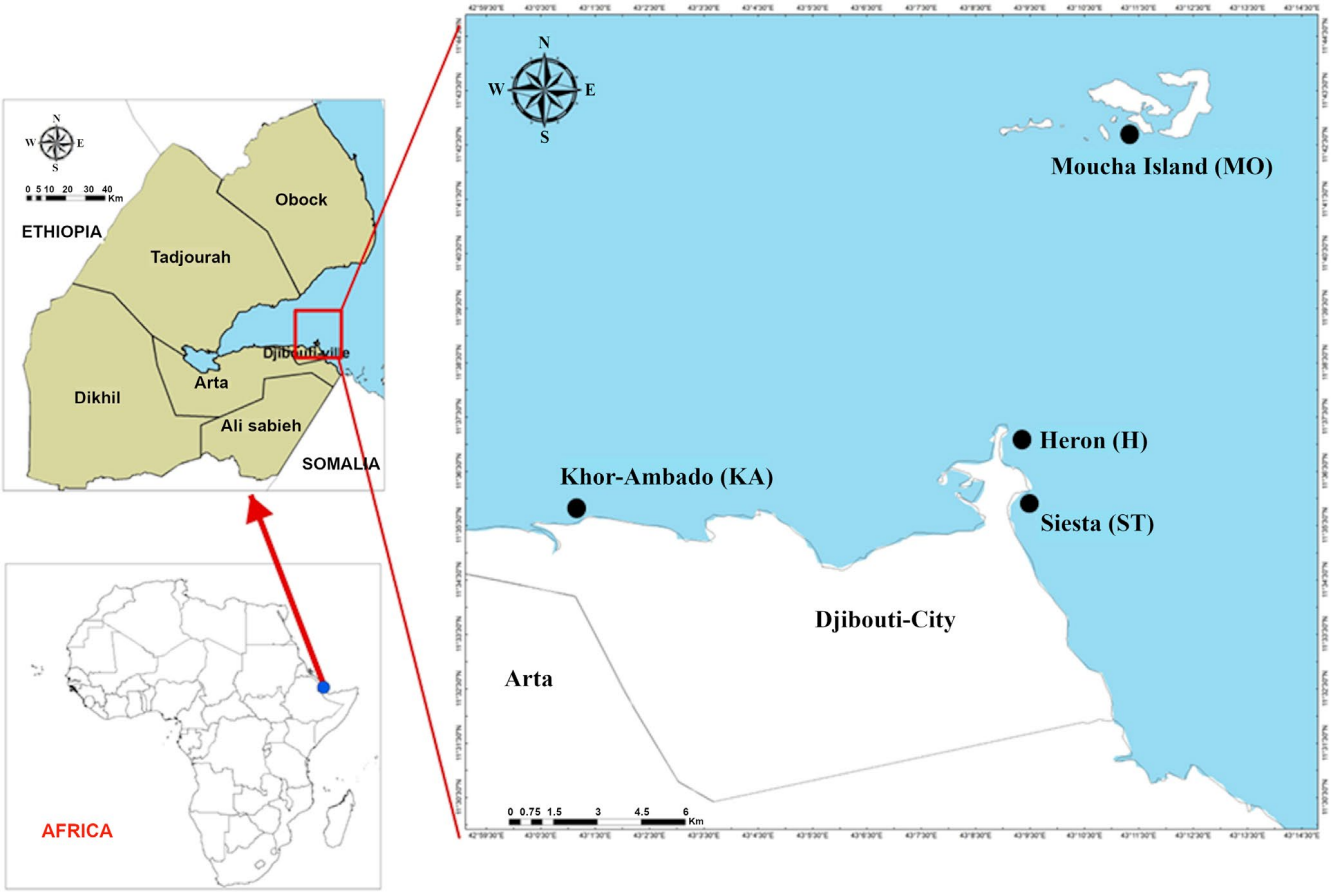
Map of the four sampling sites in Djibouti (Gulf of Tadjourah) and localization in Africa. See Table S1 for their description and GPS coordinates.

Algae were collected in the coastal area of Djibouti City at the sites Heron (H) and Siesta (ST) and two other sites, Khor‐Ambado (KA) and Moucha (MO) Island, in the southeastern Gulf of Tadjourah during the summer of 2021 (Figure 1 and Table S1). Along the Djiboutian coast, six abundant seaweed species belonging to the three taxa Phaeophyceae, Ulvophyceae, and Rhodophyta were collected at four sites (Table S2). Some species were generally distributed (*Sargassum latifolium, Padina pavonica*) while others have a more restricted distribution and are abundant in only two sites (*Sargassum ilicifolium, Turbinaria decurrens*) or in only one site (*Ulva clathrata* and *Hypnea musciformis* ). Algae were washed with seawater, then distilled water, and frozen before freeze‐drying, except *T*. *decurrens* and *S*. *ilicifolium*, which were oven‐dried at 40°C. Dried algae were ground to a fine powder and stored at 4°C until chemical extraction.

### 2.2 Preparation of Hydro‐Ethanolic Extracts and Their Total Phenolic Content (TPC)

Extractions were performed, according to the protocol of Stiger‐Pouvreau et al. (2014). For this, 15 mg of algal powder was extracted in triplicate with 1.5 mL of a mixture of ethanol: water (70:30, v:v), in the dark at 40°C under agitation at 200 rpm for 2 h. The extracts were then centrifuged at 10,000 rpm for 5 min, and the supernatant was recovered. The pellets were extracted a second time with the same solvents for 1 h; then the extracts were centrifuged, and the supernatants collected as previously. A total volume of 3 mL of supernatant was obtained for each triplicate and evaporated under reduced pressure to get the crude extracts noted E70.

The TPC was determined by colorimetric spectrophotometry using an adapted Folin–Ciocalteu test (Gager et al. 2020). Wells were filled with 20 μL of extract (2 mg mL^−1^), 130 μL of distilled water, and 10 μL of Folin–Ciocalteu reagent followed by 40 μL of Na_2_CO_3_ (200 g L^−1^). The mixture was left for 10 min at 70°C. The microplate was then placed on ice to stop the chemical reaction and the absorbance was read at 620 nm. Standard phloroglucinol (brown algae) and gallic acid (green and red algae) solutions were also submitted to this assay to obtain a calibration curve. The contents were expressed as phloroglucinol equivalent (mg PGE.g^−1^ DW) for brown seaweeds and as gallic acid equivalent (mg GAE.g^−1^ DW) for green and red seaweeds.

### 2.3 Biological Activities Associated With Hydroethanolic Extracts

#### 2.3.1 DPPH Radical Scavenging Activity

The DPPH (2,2‐diphenyl‐1‐picrylhdyrazyl) assay modified according to Gager et al. (2020) was set up to determine the radical scavenging activity of the extracts. For this method, 22 μL of extract/control was added to 200 μL of a DPPH solution (25 mg L^−1^) prepared within 24 h. After 60 min in the dark at room temperature, the absorbance was read at 540 nm. Distilled water was used as a negative control and ascorbic acid, α‐tocopherol, and 2,3‐t‐butyl‐4‐hydroxyanisole (BHA) as positive controls. The radical scavenging activity of the extracts was expressed as IC50 (in mg mL^−1^), the sample concentration that results in a 50% decrease in DPPH activity. The lower the IC50, the higher the antioxidant activity. The analyses were performed in triplicate for each extract.

#### 2.3.2 Antioxidant Activity by FRAP

The ferric reducing activity was evaluated by redox reaction between phenolic compounds in the extract and transition metal ions such as ferric ions (Gager et al. 2020). In a microplate, 25 μL of sodium phosphate buffer (0.2 mM, pH 6.6) and 25 μL of 1% potassium ferricyanide were added to 25 μL of extract/control. After homogenization, the microplates were incubated at 50°C for 20 min. The reaction was then stopped on ice. An initial absorbance measurement was performed at 620 nm after the addition of 25 μL of trichloroacetic acid and 100 μL of distilled water. Finally, 20 μL of iron chloride was added. After 10 min, the absorbance was measured at 620 nm. Ascorbic acid, α‐tocopherol, and BHA were used as positive controls. The reducing power was expressed as EC50 (mg mL^−1^). Finally, the lower the EC50 concentration, the higher the antioxidant activity.

#### 2.3.3 Oxygen Radical Absorbance Capacity (ORAC)

The ORAC test measures the capacity of antioxidants in a sample to absorb oxygen radicals. It is based on the oxidation of a fluorescent probe, fluorescein, by the addition of a free radical generator (2,2′‐azobis 2‐amidinopropane dihydrochloride, AAPH) which inhibits fluorescein over time (inducing a decrease in fluorescence over time). Antioxidant molecules present in the sample block the generation of free radicals until the sample's antioxidant activity is exhausted. To validate the assay, 25 μL of standard (Trolox) or extracts, or TPK (Potassium Phosphate Buffer) or water (negative controls), or BHA (positive control) was added to each well followed by 150 μL fluorescein (diluted 2:1 with TPK) except in wells with water. The microplate was shaded, incubated for 10 min at 37°C, and 25 μL of AAPH was added to each well to start kinetics. The results were expressed in μmol eq.trolox.mg^−1^ algal DW. The higher the percentage, the more significant the ORAC antioxidant activity.

#### 2.3.4 Pigments Extraction and Analysis

Chlorophylls and carotenoids were extracted according to Lalegerie et al. (2019). Extractions were carried out using 50 mg DW of algal powder, in the presence of 500 μL of a mixture of acetone: water (90:10), for 30 min at 4°C under magnetic agitation. After this first extraction, samples were centrifuged at 10,000 rpm for 5 min (miniSpin plus, Eppendorf) and the supernatant was collected. The remaining pellets were again mixed with 90% acetone for 4 h at 4°C. After the second extraction, samples were centrifuged, and both supernatants were combined and filtered through 0.2 μm‐pore syringe filters. HPLC analysis was performed directly after extraction on a Dionex Ultimate 3000 system equipped with a diode array detector (Thermo Scientific). Pigments were separated using an ACE C18 column (150 × 4.6 mm, 3 μm) protected with a guard column; the mobile phase consisted of (A) methanol: ammonium acetate buffer 0.5 M (80:20), (B) acetonitrile: Milli‐Q water (87.5:12.5) and (C) ethyl acetate 100%. A flow rate of 1.0 mL min^−1^ was used with the following gradient: 0 min 90:10 (A:B, v:v), 1 min 0:100 (A:B, v:v), 11 min 78:22 (B:C, v:v), 24 min 25:75 (B:C, v:v), 26 min 25:75 (B:C v:v), 27 min 100:0 (B:C, v:v), 28 min 90:10 (A:B, v:v) and 33 min 90:10 (A:B, v/v). The column temperature was set at 30°C ± 1°C, and the injector temperature was 5°C ± 1°C. Each sample was automatically diluted 3/4 with ammonium acetate buffer (0.5 M, pH 7.2), just before injection (volume of injection = 6 μL) to achieve better separation. Pigments were identified by comparison of retention times and absorption spectra with commercial standards of chlorophyll‐*a* (Sigma, USA), chlorophyll‐*c*, fucoxanthin, pheophytin‐*a*, β‐carotene, and lutein (DHI, Denmark). Pigment concentrations were then quantified using standard curves obtained through the injection of different concentrations of the standards. Only peaks with an area larger than 0.4 mAU min for β‐carotene and 1 mAU min for the other pigments were quantified. Chromeleon 7 software (Thermo Scientific Dionex, France) was used for HPLC control and data acquisition; the results are expressed as mg g^−1^ algal dry weight (% DW).

#### 2.3.5 Extraction and Determination of Carbohydrates

Carbohydrates were extracted according to DuBois et al. (1956). For this, 100 mg of algal powder was added to 1 mL of distilled water; the mixture was vortexed for 5 min and then centrifuged at 1000 rpm for 10 min. The pellet was recovered for the determination of cell‐wall bound sugars, and the supernatant was recovered for the determination of soluble sugars. In a glass tube, 400 μL of the pellet/supernatant was taken, and 400 μL of phenol (50 g L^−1^) and 2 mL of 98% sulfuric acid were added. The mixture was vortexed and left to stand for 10 min at room temperature, then the mixture was shaken for 30 min at 35°C. Then the mixture was cooled on ice. Finally, 200 μL of the mixture was placed on a microplate for absorbance reading at 492 nm. Glucose was used as the standard. The results are expressed as a percentage of carbohydrate relative to algal dry weight (% DW).

#### 2.3.6 Extraction and Determination of Mannitol

Mannitol was extracted according to Chades et al. (2018). For this, 50 mg of algal powder was extracted with 500 μL of 0.05 M hydrochloric acid at room temperature for 15 min, and then the mixture was centrifuged at 10,000 rpm for 5 min. To assay the mannitol, 10 μL of the supernatant was added to 50 μL of sodium formate (0.5 M, pH 3), 30 μL of sodium periodate (5 mM) and 30 μL of solution A (0.1 M acetylacetone +2 M ammonium acetate +0.02 M sodium thiosulfate) according to Sánchez‐Campos et al. (1998). The mixture was heated at 75°C for 6 min, then the absorbance was read at 412 nm. The standard used was mannitol (stock solution) dissolved in HCl at 0.05 M, with the range going from 0 to 1.500 nmol mL^−1^. Finally, the results were expressed as a percentage of mannitol relative to algal dry weight (% DW).

#### 2.3.7 Protein Extraction and Assay

Protein extractions and assays were determined using published procedures (Badmus et al. 2019; Smith 1985). For this, 50 mg of algal powder was extracted with 990 μL of phosphate buffer (0.2 M; pH 8) and 10 μL of EDTA (0.5 M); then, 2 μL of 2‐mercaptoethanol was added, and the mixture was stirred for 4 h at room temperature. After centrifugation for 15 min at 8000 rpm, the pellet was analyzed for insoluble proteins, and the supernatant was analyzed for soluble proteins. To perform the test, 10 μL of samples (pellet/supernatant) were mixed in a microplate with 200 μL of Bicinchoninic Acid (BCA) Protein Assay Kit (Sigma‐Aldrich, St. Louis, MO, USA), and the mixture was heated to 37°C, then cooled on ice, and finally, the absorbance was measured at 562 nm. We used two standard ranges: (1) BSA diluted in distilled water (soluble protein dosage) and (2) BSA diluted in 0.5 M NaOH (insoluble protein assay). The results are expressed as a percentage of protein relative to algal dry weight (% DW).

#### 2.3.8 Amino Acid Extraction and Assay

Amino acids were extracted according to Lalegerie et al. (2019). To do this, 50 mg of algal powder were extracted with 1 mL of phosphate buffer (0.1 M; pH 7.5). The solution was vortexed and then placed in the ultrasonic bath at 4°C for 15 min, then centrifuged at 10,000 rpm for 15 min. The supernatant was collected, and 800 μL of phosphate buffer (0.1 M; pH 7.5) was added to 200 μL of supernatant (5‐times dilution). Then in a microplate, 30 μL of diluted samples, 15 μL of citrate buffer (0.2 M; pH 4.6) and 30 μL of ninhydrin reagent (0.96% w/v, 0.054 M) were mixed and placed at 95°C for 20 min. Then the microplate was cooled for 5 min on ice, and finally, 90 μL of 60% ethanol was added, and the absorbance was read at 570 nm. We used a range of leucine heel concentrations between 0 and 1 mM (≥ 98% purity purchased from Sigma‐Aldrich (St. Louis, MO, USA)); thus, the AA content is expressed in mg leucine g^−1^ algal DW.

### 2.4 Determination of Minerals and Heavy Metals

Minerals and heavy metals were determined using the method by Waeles et al. (2015): 30 mg of dry and ground algal samples were digested in a mixture of HNO_3_ (65%) and hydrogen peroxide (30%), heated to 80°C for 3 h. The concentrations of trace metals (Fe, Mn, Cu, Zn Co), macroelements (Ca, Mg, Na, K, P), heavy metals (Cr, Ni, Cd, Pb) and metalloids (As) were then measured. The essential trace element iodine was not measured in any of the six species, as it was demonstrated that Fucales species are not rich in iodine, unlike Laminariales species (Nitschke and Stengel 2015). We therefore did not wish to look for iodine‐rich species in the panel of species studied.

Concentrations were determined by inductively coupled plasma mass spectrometry (ICP‐MSX series‐2 quadrupole; Thermo Scientific, United States; operated at the Brest Ocean Spectrometry Center), an analyzer that can detect metallic and non‐metallic elements at very low concentrations. Finally, the contents of the different elements were determined; the trace element, heavy metal, and metalloids contents were expressed as μg g^−1^ algal DW and macroelement in mg g^−1^ algal DW.

### 2.5 Recommended Dietary Allowance (RDA) of Minerals and Biochemical Composition as Well as Health Risk Assessment for Metals

The Recommended Dietary Allowance (RDA) is a set of reference values for the average daily intake level sufficient to meet the nutritional requirements of most healthy individuals. According to Directive 2008/100/EC, a significant amount of each element corresponds to more than 15% of the RDA. According to Circuncisão et al. (2018) and Nunes et al. (2017), we calculated the Recommended Dietary Allowance (RDA) for a daily portion of 8 g of seaweed, as also described by MacArtain et al. (2008) and Miyake et al. (2006) for the Asian population. This analysis focuses on minerals, proteins, and carbohydrates. A risk assessment of potential health hazards associated with consuming food products containing seaweed meal was conducted using the Target Hazard Quotient (THQ). The exposure dose for each heavy metal was estimated as follows: Exposure dose = (C × D_DW_)/Bw, where C is the concentration of the heavy metal in dry seaweed, D_DW_ is the daily dry seaweed biomass intake (8 g), and Bw is the average human body weight, taken at 70 kg, according to Chen et al. (2018). The Target Hazard Quotient (THQ) was then calculated as follows: THQ = Exposure dose/ML, where ML is the recommended limit value for human consumption for edible species.

### 2.6 Individual Blind‐Tasting Test

We organized a tasting test during which 20 people took part, all of whom were equally matched in terms of gender and over 50% of whom were under 30 (Table S3). During the test, two seaweed‐based preparations were offered to each person: a plain doughnut made with seaweed flour and a seaweed tartare made from various ingredients, including seaweed flakes (see recipe below).

**FIGURE 2:**
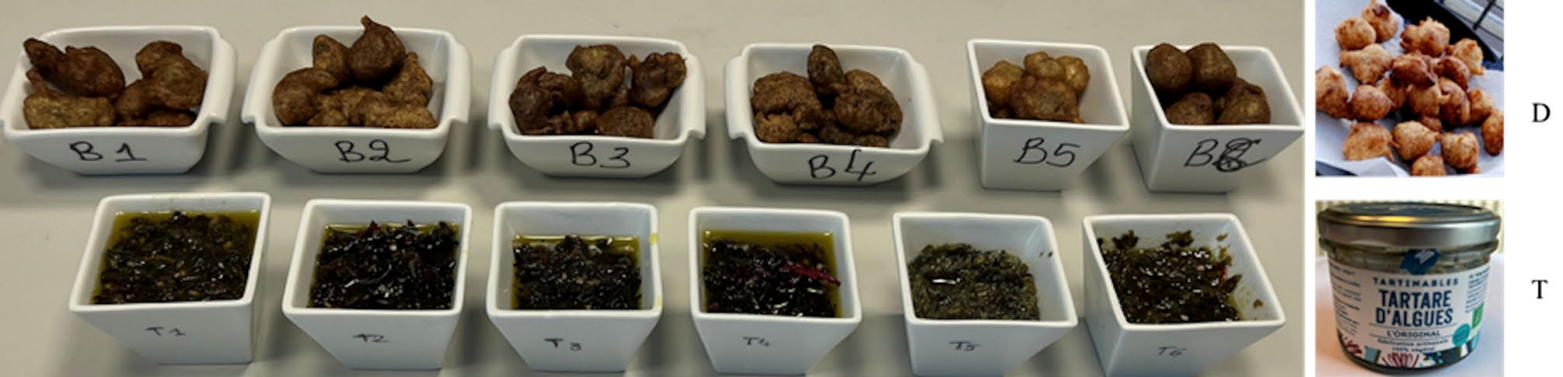
The organoleptic evaluation of doughnuts (D) and tartares (T) prepared from different seaweed species, used as flour (doughnut) or used as flakes (tartare), with a comparison with a doughnut made with 100% wheat flour and a commercial tartare.

First, we made the seaweed flour as follows: the seaweed was washed in freshwater to remove all traces of salt and then dried. They were then cut into pieces and ground into a powder. To prepare the doughnut, we used the ratio of 25 g of seaweed flour to 75 g of wheat flour, repeated five times to make enough doughnuts. These ingredients were then mixed with water and a pinch of salt and left to stand for 4 h before being fried in pre‐heated oil. Around 25 small doughnuts were prepared for each species of seaweed, allowing comparative tasting (Figure 2). As a control, we prepared a doughnut made with 100% wheat flour. For the preparation of tartares, we followed the recipe on the Food'algues website (https://foodalgues.bzh/articles/recettes/tartare‐d‐algues) without the addition of capers so as not to alter the taste. Fifteen grams of rehydrated seaweed were cut and soaked in 100 mL olive oil. Additional minor ingredients such as ginger, pepper, salt, and carrot were added. The mixture was refrigerated for 3 h. Six portions of tartare were prepared for each type of seaweed to allow comparative tasting. As a control, we used a commercial tartare (Figure 2).

For this blind test, we coded the doughnuts and tartares as follows: Doughnuts (D) and Tartares (T) and the macroalgal species were also coded as follows: *P*. *pavonica* (1), *S*. *ilicifolium* (2), *S*. *latifolium* (3), *T*. *decurrens* (4), *U*. *clathrata* (5), and *H*. *musciformis* (6).

The sensory evaluation of these food products is based on four parameters: aspect, smell, taste, and texture, with scores given for each sensation ranging from 0 to 10. The test was carried out individually and blindly, in the sense that people had in front of them batches of doughnuts and tartares coded D1 to D6 (B1 to B6 on Figure 2 for “beignet” in French) and T1 to T6, respectively. Each person completed a questionnaire individually. The data collected was processed as a radar graph, making it possible to observe and compare the different doughnuts and tartares for the four parameters (Figure 4).

### 2.7 Statistical Analysis

All analyses were conducted using Origin Pro statistical software (version 2024). Each analysis was performed three times (*n* = 3), and the measured values were reported as the mean with standard deviation. A normality analysis using the Shapiro–Wilk test was conducted, followed by a non‐parametric analysis (Kruskal‐Wallis), since the data did not meet the three feasibility criteria for the application of an ANOVA. The Pearson test was used to evaluate the correlations between the means of the biochemical compartments compared in the study. All the data showed significant differences with a risk of 5% (when *p* < 0.05).

## 3 Results

### 3.1 Phenolic Content of Species and Associated Antioxidant Activities

The average content of phenolic compounds (TPC) and their associated antioxidant activities (DPPH, FRAP, and ORAC) of the six seaweeds analyzed are presented in Table 1. The species showed significantly different TPC contents (Kruskal‐Wallis, *p* < 0.001). *Padina pavonica* had the highest TPC at 6.42 mg PGE g^−1^ DW, followed by *T*. *decurrens* at 2.31 mg PGE g^−1^ DW. *Sargassum ilicifolium* and *S*. *latifolium* have non‐significantly different TPC contents at 1.31 mg PGE g^−1^ DW and 1.15 mg PGE g^−1^ DW, respectively. *Hypnea musciformis* has the lowest TPC content at 1.04 mg GAE g^−1^ DW. According to the results, the radical scavenging activities (DPPH) vary from IC50 = 0.08 mg mL^−1^ for *P*. *pavonica* (close to positive controls) to 3.41 mg mL^−1^ of *S*. *ilicifolium* . Moreover, *Sargassum latifolium* and *T*. *decurrens* have weak and similar antioxidant activities with an IC50 of 2.50 and 2.10 mg mL^−1^ respectively. In the case of the antioxidant activity, measured using the FRAP assay (EC50), it varied between 0.03 mg mL^−1^ for *P*. *pavonica*, close to positive controls, to 1.44 mg mL^−1^ for *S*. *latifolium* . Concerning the antioxidant activity determined in ORAC units, the results varied from 7.09 mmol eq Trolox mg^−1^ from *H*. *musciformis* to 130.2 mmol eq Trolox mg^−1^ in *P*. *pavonica*, close to positive controls.

**TABLE 1:** Total phenolic content (TPC: mg PGE g^−1^ DW for brown and mg GAE g^−1^ DW for green/red seaweeds) in ethanol: water (70:30) E70 extracts from Djiboutian abundant brown, green and red seaweeds, and radical scavenging and antioxidant activities (mean ± standard deviation, *n* = 3).

Species	Site	TPC	DPPH IC50	FRAP EC50	ORAC
		mg PGE g^−1^ DW or mg GAE g^−1^ DW	mg mL^−1^	mg mL^−1^	μmol eq Trolox mg^−1^
*Padina pavonica*	KA	**6.42 ± 0.41** ^**a**^	**0.08 ± 0.01** ^**c**^	**0.03 ± 0.00** ^**e**^	**130.93 ± 0.63** ^**a**^
*Sargassum ilicifolium*	H	1.31 ± 0.02^c^	3.41 ± 0.60^a^	1.41 ± 0.32^a^	25.35 ± 1.21^e^
*Sargassum latifolium*	ST	1.15 ± 0.11^c^	2.10 ± 0.01^ab^	1.44 ± 0.25^a^	32.13 ± 1.12^d^
*Turbinaria decurrens*	MO	2.31 ± 0.31^b^	2.50 ± 0.33^ab^	1.16 ± 0.25^b^	89.40 ± 1.35^b^
*Ulva clathrata*	ST	1.70 ± 0.27b^c^	0.87 ± 0.01^bc^	0.70 ± 0.18^c^	67.35 ± 0.35^c^
*Hypnea musciformis*	KA	1.04 ± 0.08^c^	0.45 ± 0.06^bc^	0.20 ± 0.07^d^	7.09 ± 1.12^f^
**Positive controls**					
Vitamin C			**0.02 ± 0.00**	**0.02 ± 0.0**	NA
BHA			**0.02 ± 0.00**	**0.04 ± 0.0**	**170 ± 1.5**

*Note:* Different letters indicate significant differences among species at the *p* < 0.05 level. Khor‐Ambado (KA), Moucha (MO) Island, Heron (H), Siesta (ST), see Figure 1 for the localization of collection sites. The most interesting values are highlighted in bold.

Abbreviation: NA, not analyzed.

### 3.2 Pigment and Biochemical Content, and Inter‐Species Variability

**FIGURE 3:**
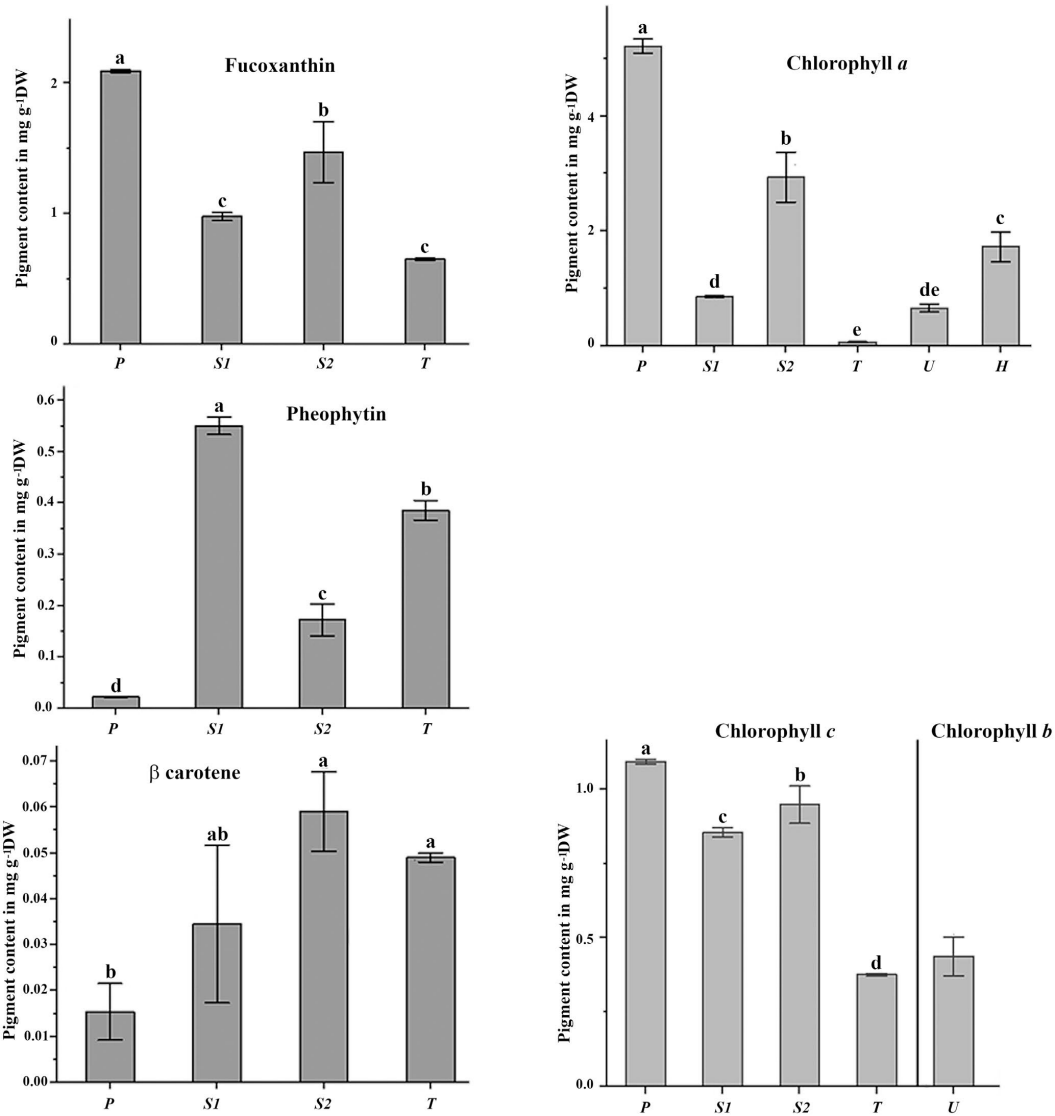
Pigment contents (in mg g^−1^ DW) of chlorophyll *a, c*, and *b*, fucoxanthin, β‐carotene, and pheophytin. Results are mean values ± standard deviation (*n* = 3). Different letters indicate significant differences among species at the *p* < 0.05 level. P: *P*. *pavonica* from KA, S1: *S*. *ilicifolium* from H, S2: *S*. *latifolium* from ST, T: *T*. *decurrens* from MO, U: *U*. *clathrata* from ST, H: *H*. *musciformis* from KA. Khor‐Ambado (KA), Moucha (MO) Island, Heron (H), Siesta (ST), see Figure 1 for the localization of collection sites.

The significant pigments in all samples were chlorophyll *a*, fucoxanthin, and chlorophyll *c* (Figure 3). Maximum chlorophyll *a* levels were observed in *P*. *pavonica* with 5.2 mg g^−1^ DW, followed by *S*. *latifolium* at 2.9 mg g^−1^ DW. Moreover, the lowest value was observed in *Turbinaria decurrens* at 0.07 mg g^−1^ DW. For chlorophyll *c*, maximum values were observed in *P*. *pavonica* with 1.1 mg g^−1^ DW (Figure 3). Lower values characterized the β‐carotene and pheophytin contents. β‐carotene ranged from 0.01 mg g^−1^ DW for *P*. *pavonica* to 0.04 mg g^−1^ DW for *T*. *decurrens* . Moreover, pheophytin ranged from 0.02 mg g^−1^ DW for *Padina pavonica* to 0.58 mg g^−1^ DW for *T*. *decurrens* . The high average of pheophytin values for *S*. *ilicifolium* and *T*. *decurrens* , respectively (0.55 and 0.58 mg g^−1^ DW), shows the degradation of chlorophyll *a* of these species during sample preparation as they were dried in an oven at 40°C. Results for fucoxanthin varied from 0.66 mg g^−1^ DW for *T*. *decurrens* to 2.1 mg g^−1^ DW in *P*. *pavonica*. The green *U*. *clathrata* had a chlorophyll *b* value of 0.45 mg g^−1^ DW.

The carbohydrate, mannitol, protein, and amino acid contents of the different macroalgal species are presented in Table 2. In our study, the total carbohydrate content showed dispersed results that ranged from 14.0% DW for *T*. *decurrens* to 39.7% DW for *H*. *musciformis* . In the case of mannitol, the contents varied from 18.6% DW in *S*. *ilicifolium* to 39.7% DW for *P*. *pavonica*. Quantitative analysis of the average total protein content ranged from 7.2% DW in *S*. *latifolium* to 12.9% DW for *H*. *musciformis* . The amino acid contents of Djiboutian abundant macroalgae vary from 10.4 mg g^−1^ DW for *S*. *latifolium* to 25.3 mg g^−1^ DW for *T*. *decurrens* . The contents were significantly different between the species (*p* < 0.05).

**TABLE 2:** Content of macroelements, trace elements, heavy metals, and metalloids.

	*Padina pavonica*	*Sargassum ilicifolium*	*Sargassum latifolium*	*Turbinaria decurrens*	*Ulva clathrata*	*Hypnea musciformis*		
Collection site	KA	H	ST	MO	ST	KA	RDI mg/Day	ML μg g^−1^
**Biochemical composition**								
Carbohydrate (%DW)	32.0 ± 1.0^b^	21.3 ± 3.7^d^	27.2 ± 1.7^c^	14.0 ± 1.2^d^	27.8 ± 0.8^c^	38.4 ± 1.1^a^	260^a^	
Mannitol (%DW)	39.7 ± 1.8^a^	18.6 ± 3.8^d^	35.4 ± 1.2^b^	20.4 ± 2.0^d^	ND	ND		
Proteins (%DW)	10.2 ± 0.5^b^	8.7 ± 2.0^c^	7.2 ± 0.7^c^	9.4 ± 0.6^b^	12.5 ± 0.9^a^	12.9 ± 0.6^a^	50^a^	
Amino acids (mg g^−1^ DW)	13.0 ± 0.6^b^	13.7 ± 0.4^b^	10.4 ± 0.2^d^	25.3 ± 1.7^a^	11.2 ± 0.8^c^	12.6 ± 2.2^bc^		
**Macroelements mg g** ^**−1**^								
P	1.09 ± 0.8^b^	0.72 ± 0.21^c^	0.92 ± 0.32^bc^	0.97 ± 0.41^bc^	1.37 ± 0.5^a^	1.50 ± 0.50^a^	700^a^	
Ca	99.0 ± 1.0^b^	39.6 ± 0.6^d^	66.1 ± 0.3^c^	22.7 ± 1.1^e^	64.3 ± 3.1^c^	140.6 ± 1.1^a^	800^a^	
Mg	16.7 ± 0.8^a^	16.7 ± 0.6^a^	1.37 ± 0.02^c^	3.2 ± 0.4^c^	13.1 ± 0.2^b^	13.3 ± 0.2^b^	375^a^	
Na	4.39 ± 1.02^b^	10.0 ± 5.7^ab^	5.67 ± 0.37^b^	3.93 ± 0.33^c^	9.34 ± 1.3^ab^	15.3 ± 2.1^a^	600^a^	
K	1.36 ± 0.82^d^	9.50 ± 0.70^a^	0.33 ± 0.15^d^	1.57 ± 0.42^c^	6.53 ± 1.50^b^	1.39 ± 0.20^d^	2000^a^	
Na/K	3.2	1.0	17.3	2.5	1.4	11.0		
Ca + Mg + Na + K	125.3	75.8	73.4	31.4	93.3	170.5		
**Trace elements μg g** ^**−1**^								
V	13.0 ± 0.8^a^	2.56 ± 0.21^d^	3.59 ± 0.52^cd^	1.35 ± 0.11^e^	4.90 ± 0.10^c^	7.41 ± 0.51^b^		
Mn	184 ± 8^a^	19.4 ± 2.1^e^	77.6 ± 1.6^c^	10.6 ± 0.2^e^	49.5 ± 1.5^d^	92.6 ± 3.1^b^	2^a^	
Fe	4080 ± 220^a^	270 ± 58^d^	674 ± 23^c^	152 ± 9^e^	2180 ± 50^b^	2340 ± 200^b^	8 (men), 18 (women) ^b^	
Co	3.97 ± 0.18^a^	0.99 ± 0.04^d^	1.79 ± 0.02^b^	0.59 ± 0.01^e^	0.85 ± 0.02^d^	1.36 ± 0.06^c^		
Cu	6.09 ± 0.50^a^	1.78 ± 0.39^c^	1.67 ± 0.04^c^	0.73 ± 0.07^d^	3.73 ± 0.10^b^	4.45 ± 0.38^b^	1^a^	
Zn	12.1 ± 0.7^b^	6.54 ± 1,32^c^	7.77 ± 0.33^c^	3.31 ± 0.42^d^	11.1 ± 0.4^b^	28.0 ± 1.7^a^	10^a^	
**Heavy metals μg g** ^**−1**^								
Cr	9.91 ± 0.55^b^	2.45 ± 0.58^c^	2.84 ± 0.25^c^	2.21 ± 0.35^c^	29.61 ± 0.96^a^	8.44 ± 0.45^b^		
Ni	8.68 ± 0.42^a^	1.25 ± 0.12^cd^	2.71 ± 0.12^bcd^	0.65 ± 0.01^d^	3.38 ± 0.18^bc^	5.24 ± 0.25^b^		
As	12.5 ± 1.29^c^	37.4 ± 2.52^b^	37.3 ± 1.38^b^	70.2 ± 3.7^a^	7.31 ± 0.44^d^	6.72 ± 0.12^d^		40^c^
Cd	1.37 ± 0.0^b^	1.25 ± 0.1^b^	1.77 ± 0.0^a^	1.00 ± 0.01^c^	0.22 ± 0.02^e^	0.65 ± 0.05^d^		3^c^
Pb	1.25 ± 0.22^c^	0.32 ± 0.12^de^	0.54 ± 0.11^d^	0.11 ± 0.01^e^	1.90 ± 0.02^b^	2.26 ± 0.21^a^		3^c^

*Note:* Values are mean ± SD standard deviation (*n* = 3). Different letters indicate significant differences among species at a risk of 5% (*p* < 0.05). RDI: Recommended dietary intake, established by the European Parliament and the Council of UE: Nutrient Reference values (NRV) for an adult. ^a,b^EuropeanCommission (2008), Skrzypczyk et al. (Skrzypczyk et al. 2023).

Abbreviations: ML, maximum limit; ND, not determined.

### 3.3 Contents of Essential Macroelements (Ca, Mg, Na, K, P) and Trace Elements (V, Mn, Fe co, cu, Zn)

The macroelement and trace element composition of the different species of abundant Djiboutian macroalgae are presented in Table 2, and their contribution to the Recommended Daily Allowance (RDA) intake is presented in Table 3. Phosphorus (P) content varied from 0.72 mg g^−1^ DW in the brown *S*. *ilicifolium* to 1.50 mg g^−1^ DW in *H*. *musciformis* red seaweed; its contribution to the RDA is low (< 1.7%). Ca varies from 22.1 to 140.6 mg g^−1^ DW from *H*. *musciformis* ; in addition, all species showed a significant RDA for Ca. Moreover, Mg level varied from 1.3 mg g^−1^ DW from *S*. *latifolium* to 16.7 mg g^−1^ DW from *S*. *ilicifolium* and *P*. *pavonica*; thus, it significantly contributed to the Mg RDA of 36%. Na levels ranged from 3.93 mg g^−1^ DW in *T*. *decurrens* to 9.50 from *S*. *latifolium* . Similarly, for K, it varied from 0.33 mg g^−1^ DW in *S*. *latifolium* to 9.50 mg g^−1^ DW from *S*. *ilicifolium* . In addition, the RDA showed that Djiboutian seaweed flour provided quantities of K and N below 15% of the RDA, showing a low contribution for a consumption of 8 g of seaweed.

**TABLE 3:** For a portion of 8 g of dried seaweed, contribution (in %) to the recommended daily allowance (RDA) in macronutrients (Na, K, Ca, Mg, P), trace elements (Fe, Mn, Cu, Zn), carbohydrate and proteins.

	*Padina pavonica*	*Sargassum ilicifolium*	*Sargassum latifolium*	*Turbinaria decurrens*	*Ulva clathrata*	*Hypnea musciformis*
**Biochemical composition**						
Carbohydrate	12	8	11	6	11	16
Proteins	20	17	14	19	25	26
**Macroelements**						
P	2	1	1	1	2	2
Ca	99	40	66	23	64	141
Mg	36	36	3	7	28	28
Na	5	13	7	5	12	20
K	0.5	3.8	0.1	0.6	2.6	0.6
**Trace elements**						
Mn	74	1	31	4	20	37
Fe (men)	408	27	67	15	218	234
Fe (women)	182	12	30	7	97	104
Cu	4.9	1.4	1.3	0.6	3	3.6
Zn	1	0.5	0.6	0.3	0.9	2.2
**Heavy metals**						
As	0.03	0.10	0.10	0.20	0.02	0.02
Cd	0.05	0.04	0.06	0.03	0.01	0.02
Pb	0.04	0.01	0.02	0.01	0.06	0.08
Overall interest	3	2	2	1	3	3

*Note:* Values in green represent a significant contribution (> 15%). The risk quotients (THQ) of heavy metals and arsenic (Cd, Pb, As) with color red indicate values to be monitored. Overall interest: semi‐quantitative score, considering nutritional benefits and risks associated with heavy metal content, with the value of 3 as the best score.

Concerning trace elements, a strong variation in Mn was observed, ranging from 10.6 μg g^−1^ in *T*. *decurrens* to 184 μg g^−1^ DW in *P*. *pavonica*. Fe content showed great variation depending on the seaweed; the content varied from 154 to 4080 μg g^−1^ DW in *T*. *decurrens* and *P*. *pavonica*, respectively. RDA values for iron are all above 15%. Regarding cobalt content, it ranged from 0.59 μg g^−1^ in *T*. *decurrens* to 3.97 μg g^−1^ DW in *P*. *pavonica*. Maximum copper and zinc values are 6.09 μg g^−1^ DW in *P*. *pavonica* and 28.0 μg g^−1^ DW for *H*. *musciformis* , while minimum values were 0.73 μg g^−1^ DW and 3.31 μg g^−1^ respectively, for *T*. *decurrens* . These results showed that the contribution of Cu and Zn to the RDA is less than 15% for all the species studied.

### 3.4 Heavy Metal and Arsenic Levels (Cr, Ni, As, Cd, Pb)

The levels of various heavy metals and arsenic, as well as the health risk assessment for heavy metals in Djiboutian algae, are presented in Tables 2 and 3. Cr contents showed an inter‐species variation from 2.21 to 29.6 μg g^−1^ in *T*. *decurrens* and *U*. *clathrata* respectively.

Ni content was highest in *P*. *pavonica* at 8.68 μg g^−1^ and lowest (at 0.65 μg g^−1^) in *T*. *decurrens* . Significant differences in arsenic levels were found for different species; As levels ranged from 6.72 μg g^−1^ in *H*. *musciformis* to 70.2 μg g^−1^ in *T*. *decurrens* . Minimum levels of metals were observed in Djiboutian abundant macroalgae; in the case of Cd, it ranged from 0.22 μg g^−1^ in *Ulva clathrata* in green seaweed to 1.73 μg g^−1^ in *Padina pavonica*. Moreover, lead levels ranged from 0.11 μg g^−1^ in *T*. *decurrens* to 2.26 μg g^−1^ in *H*. *musciformis* . The Target Hazard Quotient (THQ) was calculated for each element, as presented in Table 3.

### 3.5 Correlations Between Studied Chemical Variables

**FIGURE 4:**
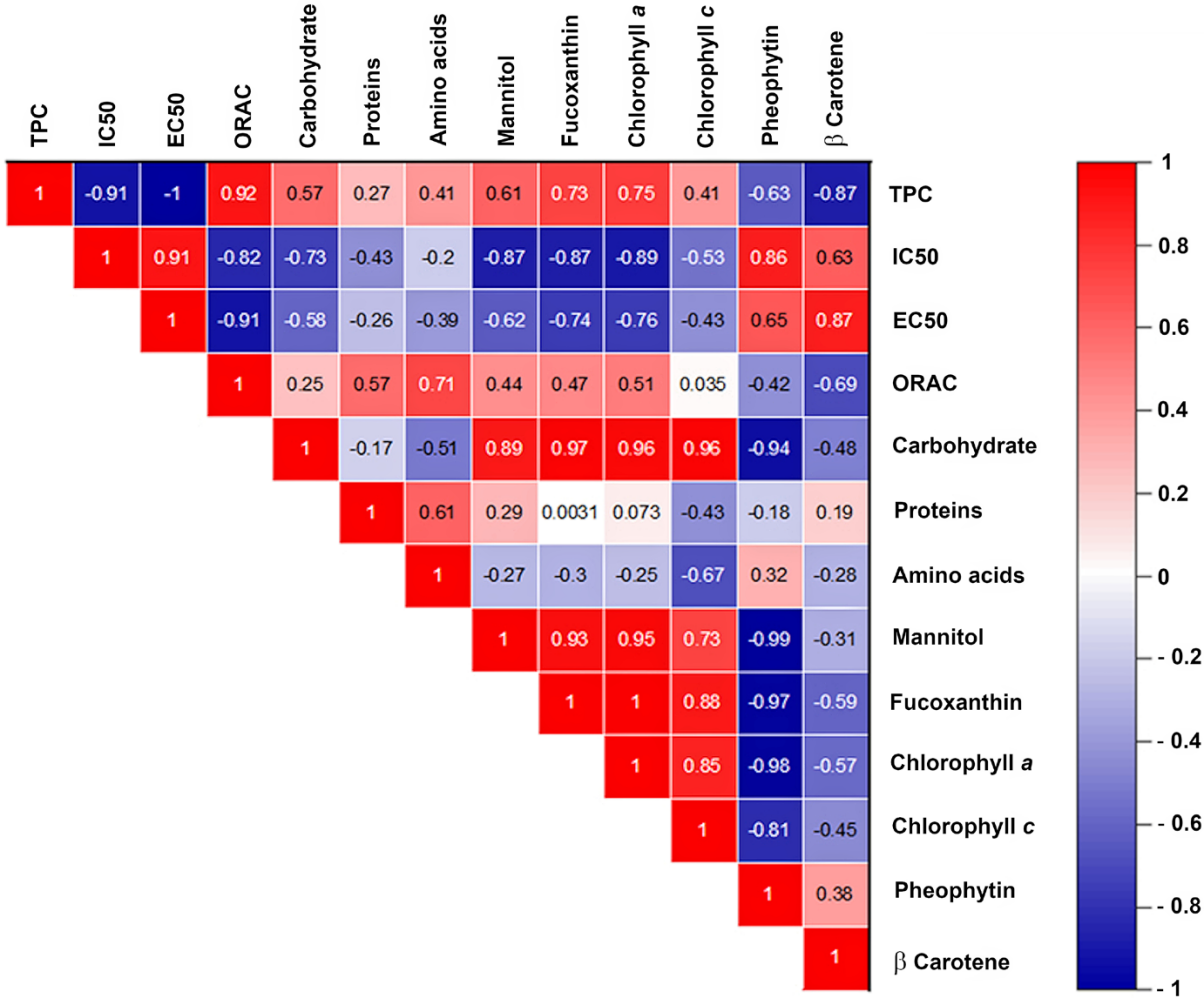
Statistical analysis using Pearson's correlation to determine relationships between different biochemical parameters *p* < 0.05.

Correlations between biochemical compositions and antioxidant activities were examined using Pearson's coefficient, as shown in Figure 4. The results revealed that total phenolic content (TPC) correlated strongly with ORAC (*r* = 0.92), as well as with IC50 (DPPH) and EC50 (FRAP) (*r* = −0.91 and *r* = −1, respectively) (*p* < 0.05). Mannitol, carbohydrates, and pigments also displayed significant correlations with antioxidant activities. Additionally, proteins and amino acids were positively correlated with each other (*r* = 0.61), while phenolic compounds were negatively correlated with pigments, that is, pheophytin and β‐carotene (*r* = −0.63 and *r* = −0.87, respectively).

### 3.6 Blind Test on Doughnuts and Tartares Using Djiboutian Macroalgae (Flour and Flakes)

**FIGURE 5:**
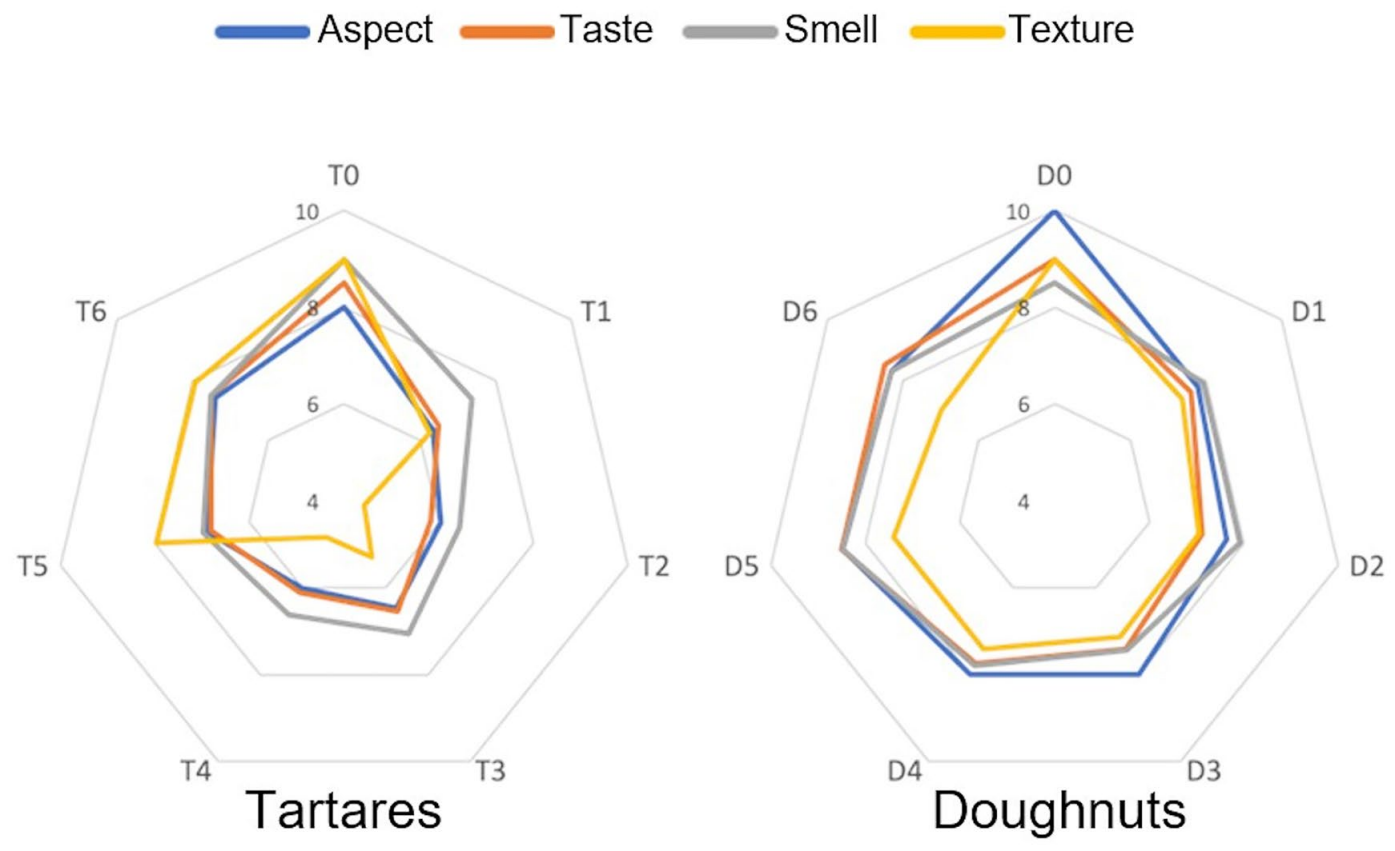
Organoleptic evaluation of Tartares (T) and Doughnuts (D), prepared from different seaweed species (from respectively flakes and flour). The data represent the average scores for each sensory parameter (aspect, smell, taste, texture) on a score scale from 0 to 10. Species were codified for the blind‐tasting test with 1: *Padina pavonica*, 2: *Sargassum ilicifolium* , 3: *Sargassum latifolium*, 4: *T*. *decurrens* , 5: *Ulva clathrata*, 6: *Hypnea musciformis* . T0 (commercial tartare) and D0 (doughnut made with 100% wheat flour) represent controls of the blind test.

Our study has shown the value of the six species for the design of flour for the cooking of doughnuts, represented as a radar graph on Figure 5. For the seaweed tartares, the results showed significant variations between species, with average scores ranging from 4.5 to 7.5 for each sensory parameter. Tartare T6 ( *H*. *musciformis* ) had the best scores in the four categories assessed. Tartare T1 (*P*. *pavonica*), on the other hand, had a notable odor and an unpleasant taste, probably due to its high content of phenolic compounds, giving it a pronounced bitterness. Tartares T2 ( *S*. *ilicifolium* ), T3 ( *S*. *latifolium* ) and T4 ( *T*. *decurrens* ) showed lower scores in terms of taste, texture, and appearance. Testers reported that these species were difficult to chew or contained large chunks of seaweed, negatively influencing their sensory evaluation. Doughnuts prepared with different seaweed flours were broadly similar, with score values above 7 (Figure 5).

Only the taste scores varied slightly, with D6 ( *H*. *musciformis* ) having a sweet taste. The D5 green algae doughnut (*U*. *clathrata*) obtained the highest scores regarding appearance, texture, and odor. All doughnuts have particular characteristics, with *Padina* producing very fine flour and a doughnut similar to those obtained with conventional flour (control). The use of an *Ulva*‐based flour gave the doughnut a slight marbled flavor.

As for the species of the genera *Sargassum* and *Turbinaria* (Sargassaceae), the flour had a coarse grain size that could be felt in each doughnut without being unpleasant to the palate. Moreover, the Sargassaceae flours gave the fritters an iodized, marine flavor. Finally, the D6 doughnut made from the red seaweed *Hypnea musciformis* flour was much appreciated for its sweet taste and identical appearance to a doughnut made from 100% wheat flour (Figure 2, top right).

## 4 Discussion

This study aims to evaluate the biochemical composition, trace elements, heavy metals, and antioxidant activities associated with polyphenols in six abundant marine macroalgae collected in the coastal waters of Djibouti (Gulf of Tadjourah), along with assessing correlations between chemical variables. We also aim to identify macroalgae with the highest levels of various nutritional components such as carbohydrates, proteins/amino acids, as well as photosynthetic pigments such as chlorophyll‐a and chlorophyll‐c/fucoxanthin, determine the daily recommended intake and risk value for heavy metals, and finally test their incorporation into two recipes, a doughnut and a tartare. Our study highlighted their potential as a source of food supplement, nutraceuticals, and ingredients for the cooking of recipes, doughnut and tartare.

### 4.1 Variability of Antioxidant Phenolic Compounds According to Species and Their Interest as Nutraceuticals

Phenolic compounds represent a crucial molecular group that plays a significant role in the antioxidant activity of tissues (Chan et al. 2007). In this study, *Padina pavonica* showed the highest TPC content in the present study (6.42 mg PGE g^−1^ DW), similar results were reported by Abdelhamid et al. (2018) who obtained a content of 7.06 mg PGE g^−1^. Others studies suggested more elevated levels, with 33.11 PGE g^−1^ DW for a methanolic extract (Matanjun et al. 2008). The lowest TPC value was recorded in the red seaweed *Hypnea musciformis* with 1.04 mg GAE g^−1^, that is, approximately 12 times lower than that reported for *H*. *musciformis* from Tunis with 12.5 mg g^−1^ according to Hmani et al. (2021). A high‐temperature extraction of *Ulva spp* (sea lettuce), an edible species in Indonesia, showed a concentration of phenolic compounds of 39 mg GAE g^−1^ DW (Pangestuti et al. 2021); this value is 39 times higher than that of *U*. *clathrata* in this present study, 1.70 mg GAE g^−1^ DW. With intermediate values, species of *Turbinaria* and *Sargassum* produced between 1.15 ( *S*. *latifolium* ) and 2.31 ( *T*. *decurrens* ) mg PGE g^−1^ DW. The significant variations in phenolic content among species harvested from different sites, as suggested in the literature, highlight the influence of several factors on phenolic compound content, such as the harvesting site, the extraction method, and the nature of the solvent (Jégou et al. 2021; D'Este et al. 2017).

In our study, we observed that *P*. *pavonica* showed the lowest IC50 for DPPH, with a value of 0.08 mg mL^−1^, which is significantly lower than the results obtained in previous work by Kosanić et al. (2019) who reported a value of 0.691 mg mL^−1^. In addition, *P*. *pavonica* also showed the lowest EC50 for FRAP and the highest ORAC antioxidant activity. This species contains a significant phenolic content that could contribute to its antioxidant properties, as shown by the findings of Sabeena Farvin and Jacobsen (2013); Zubia et al. (2008, 2020). In addition, there is a strong positive Pearson correlation (Figure 5) between total phenolic content (TPC) and radical scavenging activity IC50 (DPPH) (*r* = −0.91), antioxidant activity EC50 (FRAP, *r* = −1) and ORAC (*r* = 0.92). *Sargassum ilicifolium* and *H*. *musciformis* exhibited moderate antioxidant activities in the FRAP assay, with EC50 values of 1.41 and 0.20 mg mL^−1^ respectively (Table 1). Their phenolic contents were low, and a close Pearson correlation was observed between the FRAP EC50, chlorophyll a (*r* = −0.76), and fucoxanthin (*r* = −0.71). These compounds could be at the origin of their detected activities, as previously demonstrated by Zubia et al. (2008, 2020).

### 4.2 Biochemical Composition and Its Variability Leading to Propose Algae as Supplementation Ingredients (Daily Contributions of Proteins and Sugars)

The concentrations of all specific photosynthetic pigments in algae can vary due to various environmental factors such as light intensity, growth depth, morphological structure, and the solvent used for extraction (Lann et al. 2012; Sudhakar et al. 2013). *Padina pavonica* exhibited the highest concentration of chlorophyll *a* at 5.2 mg g^−1^, which is slightly lower than the 10.07 mg g^−1^ reported by Emam et al. (2014). *Sargassum latifolium* showed chlorophyll *a* levels 10 times higher than those reported by Ismail et al. (Ismail, El Zokm, Sikaily, et al. 2023; Ismail, El Zokm, and Miranda Lopez 2023), with 0.30 mg g^−1^. Chlorophyll *c* plays an important role in photosynthesis in algae. In this study, *P*. *pavonica* had 1.1 mg g^−1^, followed by *S*. *latifolium* with 0.9 mg g^−1^. Fucoxanthin, an abundant carotenoid with a unique chemical structure that includes an allenic bond and an acetyl functional group, shows significant potential for application as an antioxidant, anticancer, antidiabetic, anti‐obesity, and anti‐inflammatory agent (Osório et al. 2020; Sulistiyani et al. 2021). In our study, the maximum level of fucoxanthin was 2.1 mg g^−1^ DW in *P*. *pavonica*. Moreover, fucoxanthin and chlorophyll *a* showed a strong correlation with the antioxidant assays IC50/DPPH and EC50/FRAP (*r* = −0.87, −0.74) and (*r* = −0.89, −0.76), respectively, suggesting that these pigments act as powerful natural antioxidants, as described by Lourenço‐Lopes et al. (2021). These results indicate that these brown seaweeds are potential sources of pigments, leading to propose these brown algal species as potential food supplements.

In this present study, the red alga *H*. *musciformis* had the highest carbohydrate content at 38.4% DW, slightly higher than the results of Bayomy and Alamri (2024) at 23.56% DW, but slightly lower than the edible red alga *Palmaria palmata* (known with its commercial name as dulse) at 49.9% DW (Fische 2016). These differences may be due to environmental variations, as indicated by Pliego‐Cortés et al. (2020). In addition, these species showed a contribution of 11%–16% to the recommended daily intake (Table 3), indicating that the high carbohydrate content of seaweed is remarkable and that it can be a valuable source of dietary fiber for human consumption (Lafarga et al. 2020). Mannitol contents are also comparable to those of other tropical species (Zubia et al. 2008). Mannitol‐rich seaweeds could be used to a certain extent as a substitute or food additive to increase the nutritional value of foods without causing hyperglycemia, as suggested by Ladero et al. (2007).

In general, the protein content of brown seaweeds is relatively low, ranging between 3% and 15% DW (Fleurence 1999), which aligns with the present study's findings (Table 2). The highest protein concentration was observed in *H*. *musciformis* , with a content of 12.9% DW, suggesting it could contribute up to 26% of the RDA for protein. Slightly higher values, reaching 20.06% DW, have been reported by Bayomy and Alamri (2024) from samples collected in the Red Sea region of Saudi Arabia. *Turbinaria decurrens* exhibited a significant content of amino acids, indicating a high nutritional value, as suggested by El‐Shenody et al. (2019). However, these variations in the protein content of algae may be related to differences between species, the geographical location of harvesting sites, and the physical and chemical parameters of the environment (El‐Shenody et al. 2019; Ismail, El Zokm, Sikaily, et al. 2023; Ismail, El Zokm, and Miranda Lopez 2023). *Ulva clathrata* demonstrated a protein content (12.5% DW) comparable to that of *Ulva lactuca* (10%–21% DW), an edible species of the same genus referred to as the commercial name Ao‐nori, whose protein content is comparable to that of protein‐rich vegetables such as soya (Fleurence 1999). Furthermore, there is an average correlation between proteins, amino acids, and antioxidant activities (Figure 4). Seaweed meals sourced from Djiboutian species can significantly contribute to the recommended daily protein intake, ranging from 14% to 26%. Consequently, algae‐derived protein offers a sustainable and highly nutritious protein source, bypassing the need for land cultivation or freshwater irrigation (Lopez‐Santamarina et al. 2020), which would be appropriate for Djibouti, which suffers from a desertic climate.

### 4.3 The Mineral Composition of Seaweed Meal and Its Impact on Mineral Intake

Seaweeds are rich in essential minerals, but the concentration of these minerals can vary depending on the type of seaweed, its geographical origin, and environmental and physiological fluctuations (Fouda et al. 2019). The mineral and heavy metal analyses are presented in Table 2. *Hypnea musciformis* showed the highest Ca value with 140 mg g^−1^ observed in the present study. Our results are close to the values found in the edible green macroalga *Ulva lactuca* , which had a content of 182.8 mg g^−1^ (Rasyid 2017). *Padina pavonica* and *S*. *ilicifolium* had the highest Mg content with 16.7 mg g^−1^ DW. Moreover, Ca and Mg presented significant RDA values; the high Ca content may be promising for growing children, while the high Mg content is essential for regulating cardiovascular blood pressure (Circuncisão et al. 2018). The results for Na are slightly higher and K lower than those reported by Farghl et al. (2021). Algae are generally characterized by their low Na/K ratio, much lower than those found in various food products such as cheddar cheese (8.7), olives (43.6) and sausages (4.9) (Paiva et al. 2016). The Na/K ratios obtained here ranged from 1.05 to 17.8 (Table 2). It is worth noting that the World Health Organization (WHO) recommends a Na/K ratio close to 1 for a cardiovascularly healthy diet, suggesting that consuming food products with this ratio or lower could be beneficial for heart health (Blaustein et al. 2012). The total sum of essential minerals (Na + K + Ca + Mg) in mg g^−1^ dry weight in all algae species was significantly higher than the values reported for vegetables. According to Dixit et al. (2018), carrots had 32.76 mg g^−1^, green peas had 4.52 mg g^−1^, potatoes had 60.15 mg g^−1^, sweet corn had 13.47 mg g^−1^, and tomatoes had 34.29 mg g^−1^. Then, incorporating Djiboutian seaweeds into dishes or using them as a vegetable would provide essential elements for the local population.

The average value of the concentrations of trace metals measured in Djiboutian macroalgae decreased in the following order: Fe > Mn > Zn > V > Cu > Co, a similar order as according to Rao et al. (2007). In our present study, the iron levels were the most significant. Among them, *P*. *pavonica* contained the highest concentration at 4020 μg g^−1^. The study agrees with the results of Orlando‐Bonaca et al. (2021), who found that Fe in *P*. *pavonica* varied between 4000 and 3000 μg g^−1^, higher than previous results of Murugaiyon et al. (2020), which were 270 μg g^−1^. It seems that environmental conditions have a significant influence on these variations. All the seaweed species found in Djibouti contained substantial amounts of iron (Fe), ranging from 15% to 408% of the Recommended Dietary Allowance (RDA). This observation suggests that seaweeds could be a potential source of dietary iron (Fe), an essential element involved in a wide variety of metabolic processes, including respiration, energy production, and deoxyribonucleic acid (DNA) synthesis (Madkour and Rashedey 2019). The highest Mn content was observed in *P*. *pavonica*, reaching 184 μg g^−1^. This interesting result was also obtained by Orlando‐Bonaca et al. (2021) who reported similar results with 180 μg g^−1^. This significant concentration covers more than 76% of the Recommended Dietary Allowance (RDA) in using *P*. *pavonica*. This result is interesting, as Mn deficiency can impair glucose tolerance together with lipid and carbohydrate metabolism, potentially causing several human diseases (Circuncisão et al. 2018). For instance, incorporation in the diet may be justified by a strongly positive contribution to the Recommended Dietary Allowance for K, Ca, Mg, Fe, and Mn, as observed in several Chilean seaweed species (Véliz et al. 2023).

### 4.4 No Health Risk Assessment for Heavy Metal and Arsenic Levels (Cr, Ni, As, Cd, Pb) in Using Djiboutian Seaweed for Human Food

The highest levels of Cr and Ni were found in *Ulva clathrata* at 29 μg g^−1^ and in *P*. *pavonica* at 8.68 μg g^−1^ respectively. These results are consistent with those observed by Ali et al. (2017). No maximum levels (MLs) have been established for Cr and Ni in seaweed used for food or feed. However, a recommended reference dose (RfD) is available for these elements. In the various Djiboutian species, arsenic (As) levels did not reveal excessively high concentrations (above 70.2 μg g^−1^ DW), suggesting that they are not hyperaccumulators of arsenic (Brooks et al. 1977; Reeves 2006). The results of this study suggested that *P*. *pavonica* samples have significantly lower arsenic levels. This may indicate a tendency for *Padina* to accumulate less arsenic than other species, as suggested by authors who have reported values of around 1.3–1.89 μg g^−1^ for arsenic in *Padina* (Šlejkovec et al. 2006; Pell et al. 2013). Although our study focused on total arsenic, a more in‐depth analysis of As speciation, with a particular focus on inorganic arsenic (i‐As), may be required to assess potential health risks. As far as European legislation is concerned, a maximum limit of 40 μg g^−1^ total As has been set for seaweed meal, but no maximum limit has been set for seaweed used as food (Lähteenmäki‐Uutela et al. 2021). All Pb concentrations were below the maximum limit (ML) of 3.0 μg g^−1^ for lead in food supplements, as reported by Lähteenmäki‐Uutela et al. (2021). In our study, *T*. *decurrens* recorded the lowest value with 0.11 ug g^−1^, moderately similar results were recorded on the Sudanese coast with 0.66 μg g^−1^ (Ali et al. 2017). For *P*. *pavonica*, the Pb level was measured at 1.25 μg g^−1^, which is consistent with the findings of Ali et al. (2017) who reported a similar value of 1.2 μg g^−1^ for this species. In terms of Cd levels, Djiboutian algae showed slightly higher concentrations than those documented in existing literature (Ali et al. 2017; Arguelles and Sapin 2021). Variations in Cd levels among algae have been attributed to differences in storage and detoxification mechanisms and fluctuations in natural and anthropogenic sources of Cd. European legislation has set a maximum limit of 3.0 μg g^−1^ Cd for food supplements composed mainly of dried algae (Lähteenmäki‐Uutela et al. 2021; Ceva 2014). Given this regulatory threshold, the average Cd content of Djiboutian seaweeds falls below the established limit. The risk assessment conducted on the content of heavy metals and metalloids in dried seaweeds indicated that the calculated exposure dose was below the Recommended Reference Dose for As, Cd, and Pb (THQ values were < 1.0 for all elements, Table 3), thus indicating their harmlessness and possible ingestion by human populations, but obviously, continuous monitoring throughout the year may be necessary to ensure year‐round use of these algae.

### 4.5 Potential Use of Djiboutian Seaweeds for the Preparation of Various Food Products

Based on sensory evaluation, Djiboutian seaweed species were found to be particularly suitable for flour formulation to be used for the preparation of doughnuts (all species) and used as such, reduced to flakes, for the preparation of tartares, with a marked preference for tartares based on the red *Hypnea musciformis* , the green *Ulva clathrata*, and the brown *Padina pavonica* macroalgae, which offer a better taste experience. In the literature, similar results were observed for *Ulva intestinalis* , which showed high scores for biscuits made from this species (Mohibbullah et al. 2023). Other studies show the beneficial effect of incorporating seaweed into foods. Rioux et al. (2017) reviewed the use of seaweeds and companies which prepare food products with seaweeds and stipulated that because of their chemodiversity, seaweeds produce many nutritional compounds, possessing for some several functional properties that may lead to many culinary innovations. One example is the work of Rodríguez et al. (2024) who incorporated brown seawed flakes in the preparation of yoghurts (0.25% *Saccharina latissima* and 0.50% *Alaria esculenta* ) and demonstrated that this addition modified the physicochemical, functional, and sensory properties of yoghurt. Another example is the study of Hell et al. (2018) who produced a functional food by incorporating flakes of the red macroalga *Palmaria palmata* (dulse) or the brown macroalga *Saccharina longicruris* into Camembert‐type cheese. Depending on the macroalga added, this addition resulted in a higher protein/carbohydrate content for the former and a higher fiber/mineral content for the latter. Interestingly, Ngadiarti et al. (2022) demonstrated the health benefits for mice to eat cookies made with the green *Caulerpa racemosa* species (also known as seagrape) to lower blood cholesterol and glucose levels, representing a potential anti‐aging novel‐functional food.

In regard to our study, using seaweeds for the cooking of doughnuts enriched with seaweed, as healthy recognized, it could therefore be well accepted by consumers and used as functional foods on the Djiboutian market. Combining gastronomy with new seaweed‐based recipes and creating dishes in which seaweed is the main ingredient would improve the dietary diversity of the Djiboutian population. Additionally, *P*. *pavonica*, with its promising mineral content and antioxidant activities, could contribute to the Djiboutian population, which suffers from mineral and natural antioxidant deficiencies. This could lead to the development of *Padina*‐based food supplements. However, the results obtained for flakes made from *Sargassum* and *Turbinaria* on the tartare test were less significant, as testers found them difficult to swallow and presented a less appealing texture, thus ranking them in the bottom three. Similar observations were highlighted by Losada‐Lopez et al. (2021), who emphasized the food neophobia exhibited by testers when confronted with new textures and flavors of seaweed. Overall, the average of the two prepared tastings was above 5, suggesting that the acceptability of seaweed‐based dishes is quite high among potential consumers. Integrating these new recipes into the menus of avant‐garde or well‐known restaurants could also increase consumer interest in seafood products, promoting a more varied and nutritious diet.

## 5 Conclusions

This study provides a better understanding of the biochemical composition, mineral analyses, phenolic content, and associated antioxidant activities of six abundant macroalgal species from Djibouti to determine the potential use of this marine biomass in human food. *Padina pavonica* stood out for its superior antioxidant activity (DPPH, FRAP, ORAC), which could be linked to its high content of phenolic compounds and carotenoids, both beneficial for health. The red macroalga *H*. *musciformis* exhibited the highest carbohydrate content among all the species studied, giving a sweet taste to doughnuts made with *Hypnea* flour. In terms of mineral elements, *P*. *pavonica*, *S*. *latifolium* , and *H*. *musciformis* had the highest levels of certain trace elements, notably manganese, iron, and cobalt, demonstrating significant RDA, which could be beneficial for individuals with deficiencies. Arsenic levels in all the species studied were below the alert threshold. However, *P*. *pavonica* had even lower levels, compliant with European standards. The use of doughnuts and tartares made from these algae could be recommended to diversify and enhance new dishes in Djiboutian cuisine. In the context of the UN's 2030 Agenda for Sustainable Development, which aims to eradicate hunger, guarantee food security, improve nutrition, and promote sustainable agriculture, algae could represent a solution to these challenges in Djibouti. As many species meet safety criteria for heavy metal and arsenic content, their biochemical composition, which is rich in essential trace elements, makes them a valuable nutritional source. However, further research and ongoing monitoring are needed to fully assess their use and impact on human health and find appropriate recipes to introduce this new type of food to the Djiboutian population.

## Author Contributions

**Moustapha Nour:** conceptualization (equal), formal analysis (equal), investigation (equal), methodology (equal), writing – original draft (equal), writing – review and editing (equal). **Valérie Stiger‐Pouvreau:** conceptualization (lead), data curation (equal), formal analysis (equal), funding acquisition (lead), investigation (equal), methodology (lead), project administration (equal), supervision (lead), validation (equal), writing – review and editing (lead). **Abdourahman Daher:** conceptualization (equal), funding acquisition (equal), project administration (equal), supervision (equal). **Solène Connan:** data curation (supporting), formal analysis (equal), investigation (equal), methodology (lead), writing – review and editing (equal). **Ahmed Ali:** investigation (supporting). **Louna Marchand:** investigation (supporting). **Matthieu Waeles:** data curation (equal), formal analysis (equal), investigation (equal), methodology (equal), writing – review and editing (equal). **Sylvain Petek:** conceptualization (equal), formal analysis (equal), funding acquisition (lead), methodology (equal), project administration (equal), supervision (lead), writing – review and editing (equal).

## Conflicts of Interest

The authors declare no conflicts of interest.

## Supporting information


**Table S1:** fsn370956‐sup‐0001‐Tables.docx.

## Data Availability

The data sets generated during and/or analyzed during the current study are available from the corresponding author upon reasonable request.
