# Supplementary material for: Nutritional Value and Antioxidant Potential of Djiboutian Abundant Seaweeds, With Their Food Applications in Doughnut and Tartare
Source: Food Sci Nutr. 2025 Sep 16;13(9):e70956. doi: 10.1002/fsn3.70956 (PMC12440808; doi:10.1002/fsn3.70956)
Supplement: Supplementary file 1 — Table S1: fsn370956‐sup‐0001‐Tables.docx. [file FSN3-13-e70956-s001.docx]

Supporting information

Nutritional value and antioxidant potential of Djiboutian abundant seaweeds, with their food applications in Doughnut and Tartare

Moustapha Nour ^a, b,*^, Valérie Stiger-Pouvreau ^b^, Abdourahman Daher ^a^, Solène Connan ^b^, Ahmed Ali ^a^, Louna Marchand ^b^, Matthieu Waeles ^b^, Sylvain Petek ^b^

***^a^*** *Centre d'Études et de Recherche de Djibouti, Institut des Sciences de la Vie ISV, Route de l'aéroport, Djibouti*

***^b^*** *Univ Brest, IRD, CNRS, Ifremer, LEMAR, IUEM, F-29280 Plouzane, France*

* Corresponding author at: *Centre d'Études et de Recherche de Djibouti, Institut des Sciences de la Vie ISV, Route de l'aéroport, Djibouti*

E-mail address: [moustapha.nour17@gmail.com](mailto:moustapha.nour17@gmail.com)

**Table S1** Sampling locations from west from east around Djibouti city (Djibouti, Gulf of Tadjourah)

| **Site name** | **Code** | **Latitude** | **Longitude** | **Morphology of the site, environment, substrate** |
| --- | --- | --- | --- | --- |
| Siesta | ST | 11°36.070'N | 43°09.554'E | Flat bottom, bay bottom close to the city, sand |
| Heron | H | 11°37.433'N | 43°09.217'E | Flat bottom, bay bottom close to the city, sand |
| Khor-Ambado | KA | 11°35.741'N | 43°01.322'E | Medium slope, fringing reef, coral |
| Moucha Island | MO | 11°43.370'N | 43°10.638'E | Flat bottom, coastal beach, coral |

**Table S2** List of species and distribution according to the sites studied

| **Family** | **Genus** | **species** |  |  |  |  |
| --- | --- | --- | --- | --- | --- | --- |
|  |  |  | **KA** | **H** | **ST** | **MO** |
| **Brown seaweeds** |  |  |  |  |  |  |
| Dictyotaceae | *Padina* | *P. pavonica* | ✓ |  | ✓ | ✓ |
| Sargassaceae | *Sargassum* | *S. ilicifolium* |  | ✓ |  |  |
| Sargassaceae | *Sargassum* | *S. latifolium* |  |  | ✓ | ✓ |
| Sargassaceae | *Turbinaria* | *T. decurrens* |  | ✓ |  | ✓ |
| **Green seaweeds** |  |  |  |  |  |  |
| Ulvaceae | *Ulva* | *U. clathrata* |  |  | ✓ |  |
| **Red seaweed** |  |  |  |  |  |  |
| Cystocloniaceae | *Hypnea* | *H. musciformis* | ✓ |  |  |  |

**Table S3** Information about people who took part in the taste test

| Gender | Nb | % |
| --- | --- | --- |
| Male | 10 | 50 |
| Female | 10 | 50 |
| Age (Years) |  |  |
| 19-30 | 11 | 55 |
| 31-40 | 5 | 25 |
| 41-50 | 2 | 10 |
| 51-60 | 2 | 10 |
